# Effects of low-level laser therapy in adults with rheumatoid arthritis: A systematic review and meta-analysis of controlled trials

**DOI:** 10.1371/journal.pone.0291345

**Published:** 2023-09-08

**Authors:** Ingrid Lourinho, Tamara Sousa, Roger Jardim, Ana Carolina Pinto, Natália Iosimuta

**Affiliations:** 1 Master Student, Post-Graduation of Health Science—PPGCS, Federal University of Amapá - UNIFAP, Macapá, AP, Brazil; 2 Researcher at Centro Iberoamericano Cochrane—Biomedical Research Institute Sant Pau (IIB Sant Pau), Barcelona, Spain; 3 Cochrane Brazil, Center for Evidence-Based Health Studies and Health Technology Assessment, Department of Emergency Medicine, Post -Graduation in Evidence-Based Health Program, Federal University of São Paulo, São Paulo, Brazil; 4 Teacher of Physiotherapy Course, Post-Graduation of Health Science–PPGCS, Federal University of Amapá - UNIFAP, Macapá, AP, Brazil; Massachusetts General Hospital, UNITED STATES

## Abstract

Rheumatoid arthritis (RA) is an inflammatory, systemic and chronic disease that mainly affects the joints. It is characterized mainly by pain, edema and joint stiffness, which can lead to significant loss of functional capacity and quality of life. Several physical therapy resources are used in the treatment of AR, such as low-level laser therapy (LLLT) and its analgesic and anti-inflammatory effects. However, the efficacy of LLLT in AR is still controversial. The objective of this study is to evaluate the efficacy of low-level laser therapy in adults with RA. Methods and findings: We searched MEDLINE, EMBASE, CENTRAL, PEDro, LILACS, IBECS, CUMED, SCIELO and ClinicalTrials.gov. Two researchers independently selected studies, extracted data, evaluated the risk of bias and assessed the certainty of evidence using GRADE approach. Disagreements were resolved by a third author. Meta-analyses were performed. Currently available evidence was from 18 RCTs, with a total of 793 participants. We found low-quality evidence suggesting there may be no difference between using infrared laser and sham in terms of pain, morning stiffness, grip strength, functional capacity, inflammation, ROM, disease activity and adverse events. The evidence is very uncertain about the effects of red laser compared to sham in pain, morning stiffness. The evidence is also very uncertain about the effects of laser acupuncture compared to placebo in functional capacity, quality of life, range of motion and inflammation. Conclusions: Thus, infrared laser may not be superior to sham in RA patients. There is insufficient information to support or refute the effectiveness of red laser, laser acupuncture and reflexology for treating patients with RA.

## Introduction

Rheumatoid arthritis (RA) is a disease of unknown etiology, characterized by systemic changes and inflammation of the synovial tissue of the joints, in the cartilage and bones. The prevalence of RA between the years of 1980 and 2018 is 0.46% [1, 2]. Commonly affected joints include the peripheral synovial joints, such as metacarpal phalanges, ankles and wrists. However, there may also be involvement of the knee, shoulders, elbows and hips [3].

These impairments are accompanied by symptoms such as pain, morning stiffness and reduced range of motion, resulting in limitations in activities of daily living, self-care, work and leisure [4]. In addition, as it is a disease that has no cure, RA needs long-term pharmacological treatment for its control. However, its effects are tempered by the risk of adverse events. Non-pharmacological treatments are also needed to prevent joint deformities, which leads to high socioeconomic costs for the patient, the family and the health system [5, 6].

Therefore, physiotherapy plays an important role in the treatment of RA, as it includes several therapeutic modalities that aim at improving functional capacity and at minimizing the impact of the disease on the patients’ quality of life. Among the approaches, the electrophysical agents, low-level laser therapy (LLLT), has gained increasing recognition for having physiologic effects, mainly mediated by photochemical actions at the cellular level that promote an increase in tissue microcirculation and may lead to anti‐inflammatory and analgesic benefits [7–11]. However, its effects in patients with RA are controversial.

A previous systematic review suggests that LLLT can be considered to relieve pain and functional stiffness in RA [12]. However, several studies have been published since then, and this new evidence has not been summarized. Therefore, the aim of this study was to assess the efficacy of LLLT in adults with RA.

## Methods

### Data sources and searches

On July 6^th^, 2022, we searched MEDLINE, Cochrane Central Register of Controlled Trials (CENTRAL), Embase, Latin American and Caribbean Health Sciences Literature (LILACS), IBECS, CUMED, Physiotherapy Evidence Database (PEDro) and Scientific Electronic Library Online (SciELO) using relevant descriptors and synonyms, adapting the search to the specifications of each database (S1 Table). To find additional studies, we also searched the Clinicaltrials.gov and searched the reference lists of included studies. We applied no language or date restrictions.

### Study selection

We included randomized clinical trials evaluating the effects of continuous low-level laser therapy or pulse beam (classes III—ranging from 632.8–1000 nm) [9, 12] in patients with RA diagnosed by American College of Rheumatology (ACR) criteria [13, 14], ACR/European League Against Rheumatism (EULAR) [15] working group or using any validated classification criteria. We used Rayyan app (https://www.rayyan.ai/) to screen the titles and abstracts of search results, retrieve the full-text reports of all potentially eligible studies and select them for inclusion [16]. Studies were included if they randomized adult participants with rheumatoid arthritis to receive LLLT versus any other treatment, sham, or no treatment, and compared its effects considering the following outcomes: pain, functional capacity, adverse events, inflammation, disease activity, range of motion, morning stiffness, muscle strength or quality of life.

### Data extraction and quality assessment

We performed this systematic review following the Cochrane Handbook recommendations [17] and reported it in accordance with the Preferred Reporting Items for Systematic Reviews and Meta-Analysis (PRISMA) recommendations (S2 Table). We registered this systematic review protocol in the International Prospective Register of Systematic Reviews (PROSPERO: CRD42020158163). To extract data from included studies, we used a predefined form. We collected detailed information on the populations, interventions, comparisons, outcomes, funding sources and conflicts of interest. We attempted to contact the authors of included studies to check missing details. We used the Cochrane risk-of-bias tool to assess the risk of bias inherent to each outcome of the included studies [17, 18]. We then assessed the certainty (quality) of body of evidence using the Grading of Recommendations Assessment, Development and Evaluation (GRADE) (S3 Table) [17]. Two review authors (INLA and NCRI) independently selected studies, extracted data, assessed the risk of bias and the certainty of evidence. We resolved disagreements through discussion or when required by consulting a third author (ACPNP).

### Data synthesis and analysis

After extraction, we analyzed the data using *Review Manager 5*.*4*.*1*. We analyzed dichotomous data as risks ratios (RRs), and continuous data as mean differences (MDs) or standardized mean differences (SMDs). If treatments, participants, and the underlying clinical question were similar enough for pooling to make sense, we undertook meta-analyses using a random-effects model. We used the I^2^ statistics to measure heterogeneity in each analysis. We planned to carry out subgroup analyses considering age, treatment duration and LLLT parameters. We also planned to perform sensitivity analyses by exploring the influence of high risk of bias studies on treatment effects [17].

## Results

### Search strategy and selection of the studies

**Fig 1:**
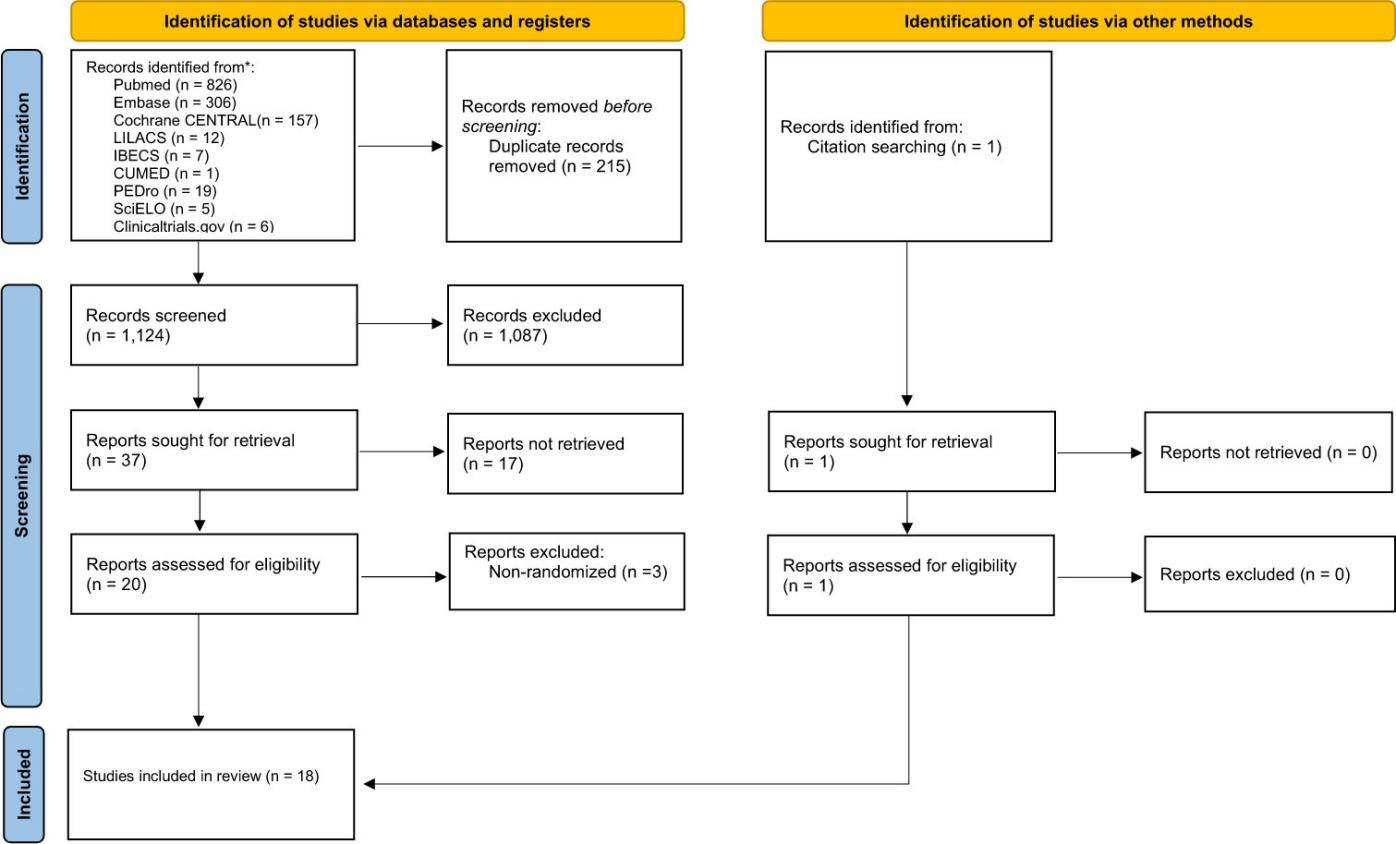
Flow diagram.

Our search strategy yielded a total of 1,339 records. After removing duplicated records, we examined 1,124 titles and abstracts. After screening titles and abstracts, 1,087 studies were excluded for not being directly related to the review question and retrieved 38 full-text reports (37 found through database and registers search and one through citation searching) for further scrutiny. Of those, 17 reports had insufficient data to include or exclude. As recommended by Cochrane Handbook [19], we did not exclude these studies, and when possible, we contacted authors for additional information. As no answers were received up to the date of submission of this paper, these studies were classified as studies awaiting classification and details on these reports are provided in S4 Table. To retrieve additional data, we also searched trial registers, such as Clinicaltrials.gov [20]. However, no additional trials were found. We excluded three reports [21–23] because they were not randomized. Finally, we included 18 studies in this review. The flow diagram can be consulted in Fig 1.

#### Included studies

We included 18 RCTs with a total of 793 participants. The included studies had intervention durations ranging from 4 weeks and 6 months.

The following comparisons were found:

1. Infrared laser versus sham (10 studies; 366 participants) [24–33]
2. Red laser versus sham (two studies; 89 participants) [34, 35];
3. Infrared laser versus laser acupuncture (one study; 40 participants) [36];
4. Laser acupuncture versus reflexology (one study; 30 participants) [37];
5. Laser acupuncture + teletherapy + methotrexate versus teletherapy + methotrexate (one study; 60 participants) [38];
6. Laser acupuncture + teletherapy versus teletherapy (one study; 60 participants) [39];
7. Infrared laser + red laser versus sham laser + naproxen (one study; 34 participants) [40];
8. Infrared laser + red laser versus naproxen (one study; 22 participants) [40];
9. Infrared laser + methotrexate + Non-steroidal anti-inflammatory drugs (NSAIDs) on demand versus methotrexate + NSAIDs on demand (one study; 114 participants) [41].

More details can be found in Table 1 and laser therapy parameters used in each study are detailed in Table 2. About the information not described in the included studies, we contacted the corresponding authors of included studies and asked whether they could provide information on those parameters, but no answer was received up to the date we submitted this article.

**Table 1:** Characteristics of randomized controlled studies included.

Study, year, location	Participants	Time since diagnosis	RA Severity	Sample size (n)	Age (Years) mean	Intervention	Time of analysis	Outcomes	Intergroup Results p-value
**1.Walker et al (1987); California** [35]	RA diagnoses according to ACR	ND	ND	I: 34 C: 38	I: 61,5 C:60	I: LLLT C: Sham	BL and on 10 weeks	Pain (VAS)	p = 0.07
**2.Adly et al (2017); Egypt** [37]	Patients fom Kasr El Ainy Medical School and National Institute of Laser Enhanced Sciences, Cairo University, Egypt. (Diagnosis criteria were not described)	ranging from 7 months to 10 years	ND	I: 15 C: 15	I: 61.83 C: 63.2	I: acupunture laser C:Reflexology	BL and on 4 weeks	Functional Capacity (HAQ) QoL (RAQoL) ROM of DFF, PFF, WF, WE and WA (goniometer) Inflammation (IL-6)	p > 0.05 p > 0.05 p < 0.05 p < 0.05
**3.Miyagi et al (1989); Japan** [31]	RA diagnoses according to ACR	I: 12.8 years C: 10.1 year	ND	I: 33 C: 35	I: 56.8 C: 54.5	I: LLLT C: Sham	BL and on 5 weeks	Pain (points scale) Functional Capacity (15 min of walk) Grip Strength Morning stiffness (min) Inflammation	p > 0.05 p < 0.01 p > 0.05 p > 0.05 p > 0.05
**4.Hall et al (1994); England** [27]	RA patients with synovitis in MCP and PIP joints (Diagnosis criteria were not described)	I:12.2 years C:9.3 years	ND	I: 20 C: 20	I: 67.1 C: 60.9	I: LLLT C: Sham	BL and on 4 weeks; follow-up of 1 and 3 months.	Pain (VAS) Functional Capacity (HAQ) Grip Strength ROM (goniometer) Inflammation	ND
**5.Meireles et al (2010); Brazil** [30]	RA diagnoses according to ACR	ND	ND	I: 41 C: 41	I: 52.44 C: 53.17	I: LLLT C: Sham	BL and on 8 weeks	Pain (VAS)—(RH/LH) Functional Capacity (HAQ) Morning stiffness (VAS) - (RH/LH) Grip Strength—(RH/LH) (dynamometer) ROM (goniometer) Inflammation (likert scale 1–5)	p = 0.16 / p = 0.15 p = 0.69 p = 0.27 / p = 0.06 p = 0.01 / p = 0.45 p = N.R. p = 0.01
**6.Palmgren et al (1989); Denmark** [32]	RA (Diagnosis criteria were not described)	ND	ND	I: 19 C: 16	I: (F: 61.1/ M: 66.0) C:(F: 57.5/ M: 8.0)	I: LLLT C: Sham	BL and on 4 weeks	Pain (VAS) Grip Strength (standardized balloon) Morning stiffness	ND
**7.Silva et al (2009); Brazil** [33]	Acute RA patients in MCP and PIP joints (Diagnosis criteria were not described)	ND	ND	I: 5 C: 5	I: 56,6 (19,9) C: NR	I: LLLT C: Sham	BL and on 5 weeks	Pain (MPQ)	p = 0.84
**8.Ekim (2007); Turkey** [24]	patients with RA and Carpal tunnel syndrome (Diagnosis criteria were not described)	I: 5.2 years C:5 years	ND	I: 10 C: 9	I: 48 (11) C: 55 (6)	I: LLLT C: Sham	BL and on 10 days, and follow-up in 3 months	Functional Capacity (Functional Status Scale) Grip Strength (dinamometer)	ND
**9.Johannsen et al (1994); Denmark** [29]	RA patients with I and II functional level according to steinbrocker (Diagnosis criteria were not described)	ND	ND	I: 10 C: 12	I:59 C:62	I: LLLT C: Sham	BL and on 4 weeks	Pain (1–12 score range) Grip Strength (dinamometer) Morning stiffness (ordinal scale 0–2) Inflammation (CPR)	ND
**10.Bliddal et al (1987); Denmark** [34]	Acute RA patients in MCP joints (Diagnosis criteria were not described)	ND	ND	I: 9 C: 8	I: 57 (41–79) C: ND	I: LLLT C: Sham	BL, 3 weeks, and follow-up in 4 weeks	Pain (VAS) Morning stiffness	p < 0.05 (on 3 weeks) p > 0.05
**11.Goats et al (1996); Scotland** [25]	Acute RA patients from Gartnavel General Hospital (Diagnosis criteria were not described)	I:7.5 years C:9.8 years	ND	I: 25 C:10	I: 57 (14) C: 64 (8)	I: LLLT C: Sham	BL, 1^st^, 3^rd^ and 6^th^ months.	Pain (VAS) Functional Capacity (HAQ) Morning stiffness (hours) Inflammation (CRP) ROM (goniometer) Disease activity level (RAI)	ND
**12.Adly et al (2021A);** **Egypt** [36]	RA diagnosis according to ACR and EULAR	ND	ND	I: 20 C:20	I: 70.9 C: 70.3	I: LLLT C: Laser acupuncture	BL, 12 weeks	Functional Capacity (HAQ) QoL (RAQoL) Inflammation (IL-6)	p > 0.05 p < 0.05 P > 0.05
**13.Adly et al (2021B);** **Egypt** [42]	RA diagnosis according to ACR and EULAR	ND	ND	I: 30 C:30	E: 68.8 C:69.1	I: Laser acupuncture + teletherapy (AEV) C: teletherapy (AEV)	BL, 4 weeks	Functional Capacity (HAQ) QoL (RAQoL) Inflammation (CRP/IL-6)	p < 0.05 p < 0.05 p < 0.05/ p < 0.05
**14.Muhamed et al (2021);** **Iraq** [40]	RA diagnosis according to ACR	ND	ND	I: 12 C1: 12 C2:10	Mean age of 49.6	I: LLLT (red +infrared) C1: sham LLLT + naproxeno C2: naproxeno	BL, 7 weeks	Pain (VAS) Morning stiffness DAS28 Inflammation (CRP)	p = 0.01 p = 0.05 p = 0.02 p > 0.05
**15. Adly et al., (2022);** **Egypt** [38]	RA diagnosis according to ACR and EULAR	ND	ND	I:30 C:30	I: 68.87 C: 69.13	I: Laser acupuncture + teletherapy + methotrexate C: teletherapy +methotrexate	BL, 4 weeks	Functional Capacity (HAQ) QoL (RAQoL) Inflammation (CRP/IL-6)	p > 0.05 p > 0.05 p < 0.05/ p < 0.05
**16. Goldman et al. (1980);U.S.A** [26]	RA classified by American Rheumatism Association criteria	Ranging from 1 year to 26 years	ND	I: ND C: ND A: 30	I: ND C: ND	I: LLLT C: Sham	BL, 10 weeks, and 3 months	Erythema Pain Flexion Grip strength PIP Range of motion MCP flexion	Significant Significant Non significant Significant Non significant Non significant
**17. Heussler et al., (1993);Australia** [28]	RA and bilateral involvement of their MCP and PIP joints (Diagnosis criteria were not described)	Ranging from 2 year to 14 years	ND	I: 25* C: 25*	I: ND C: ND	I: LLLT C: Sham	BL, 5 weeks	Pain morning stiffness Swollen ROM Grip strength Inflammation (CRP) Adverse effects	Non significant Non significant Non significant Non significant Non significant Non significant Non significant
**18. Zhuravleva et al., (2021);Russian** [41]	RA patients (Diagnosis criteria were not described)	ND	ND	I: 57 C: 57	I: ND C: ND	I: LLLT + methotrexate + NSAIDS C: methotrexate + NSAIDS	BL, 6 months	Pain morning stiffness N of patients needing NSAIDs	MD -0.51 (IC 95% -0.60 to -0.42)** MD -17.8 (IC 95% -19.67 to -15.93)** RR 2.1 (IC 95% 1.09 to 4.05)**

RA, rheumatoid arthritis; ACR: American College of Rheumatology; I: intervention group; C: control group; A: all; BL: baseline; HAQ: health assessment questionnaire; QoL: quality of life; RAQoL: rheumatoid arthritis quality of life; ROM: range of motion; DFF: dorsiflexion of foot; PFF: plantar flexion of foot; WF: wrist flexion; WE: wrist extension; WA: abduction wrist; VAS, visual analogue scales MCP, metacarpophalangeal joint; PIP, proximal interphalangeal joint; IL-6: interleukine 6; CRP: C-reactive protein; RAI: Ritchie Articular Index; EULAR: European Alliance of Associations for Rheumatology; DAS28: Disease activity score; LLLT = Low-level laser therapy; AEV: aerobic and virtual exercises; NSAIDS: Non-steroidal anti-inflammatory drugs; Ga-Al-Ar: gallium-aluminium-arsenate; ND: not described

*hands were randomized

**significant difference

**Table 2:** Low-level laser therapy parameters of included studies.

Studies	laser type	Wavelength (nm)	Fluency J/cm^2^	Power(mW); time(s)	Irradiance (W/cm2)	Number of points; number of sessions	Energy (J)
**1.Walker et al (1987)**	AlAs He-Ne	632.5; red	0.0075	1; 30	47.7	3 (radial, median and saphenous nerve on each painful joint); 3 sessions/ week	ND
**2. Adly et al (2017)**	GaAlAs	904; infrared	ND	100; ND	100	ND; 3 sessions/ week	4
**3. Miyagi et al (1989)**	GaAlAs	830; infrared	ND	20;30	ND	6 (the knee joint; 2 sessions/ week	ND
**4. Hall et al (1994)**	GaAlAs	820; infrared	3.600	40;90	ND	4 on each MCP and PIP joints; ND	3.6
**5. Meireles et al (2010)**	GaAlAs	785; infrared	3	70; ND	ND	14; 2 sessions/ week	ND
**6. Palmgren et al (1989)**	GaAlAs	820; infrared	3.58	15;60	ND	8; 3 sessions/ week	ND
**7. Silva et al (2009)**	Infrared	904: infrared	ND	8; 195	ND	ND; 2 sessions/ week	1.55
**8. Ekim et al (2007)**	GaAlAs	780; infrared	ND	50; 600	ND	5; 5 sessions/ week	7.5
**9. Johannsen et al (1994)**	GaAlAs	830; infrared	ND	21; ND	ND	4 on two MCP joints; 3 sessions/ week	2,9
**10. Bliddal et al (1987)**	He-Ne	633; red	6	10;300	ND	ND; 3 sessions/ week	ND
**11. Goats (1996)**	GaAlAs	660–950; infrared	8.1	940;240	ND	5; ND	ND
**12. Adly A et al (2021)**	Infrared	I:904; infrared C: 904 laser pucture;infrared	I:20.1C:4	I:500;30 C:100:40	I:650 C:100	I:3 C:7	I:ND C: 4
**13. Adly B et al (2021)**	Infrared	808; infrared	7.5	ND;60	100	5;ND	ND
**14. Muhamed et al (2021)**	GaAlAs; He-Ne	830; infrared 632,8; red	ND ND	7.3; ND 300; ND	ND ND	ND; 3 sessions/ week	ND
**15. Adly A et al (2022)**	Infrared	808; infrared	7.5	100;60	100	4; 6 sessions/ week	ND
**16. Goldman et al. 1980**	Infrared	1060; infrared	15–25	ND; 0,3	ND	MCP and PIP, ND	ND
**17. Heussler et al., 1993**	Infrared	820; infrared	12	50; ND	ND	MCP and PIP, ND	ND
**18. Zhuravleva et al., 2021**	Infrared	890; infrared	ND	5000;60–120	ND	ND;ND	ND

He-Ne: helium-neon; GaAlAs: gallium aluminum arsenide; AlAs: aluminum arsenide; ND: not described; MCP, metacarpophalangeal joint; PIP, proximal interphalangeal joint

#### Risk of bias in included studies

**Fig 2:**
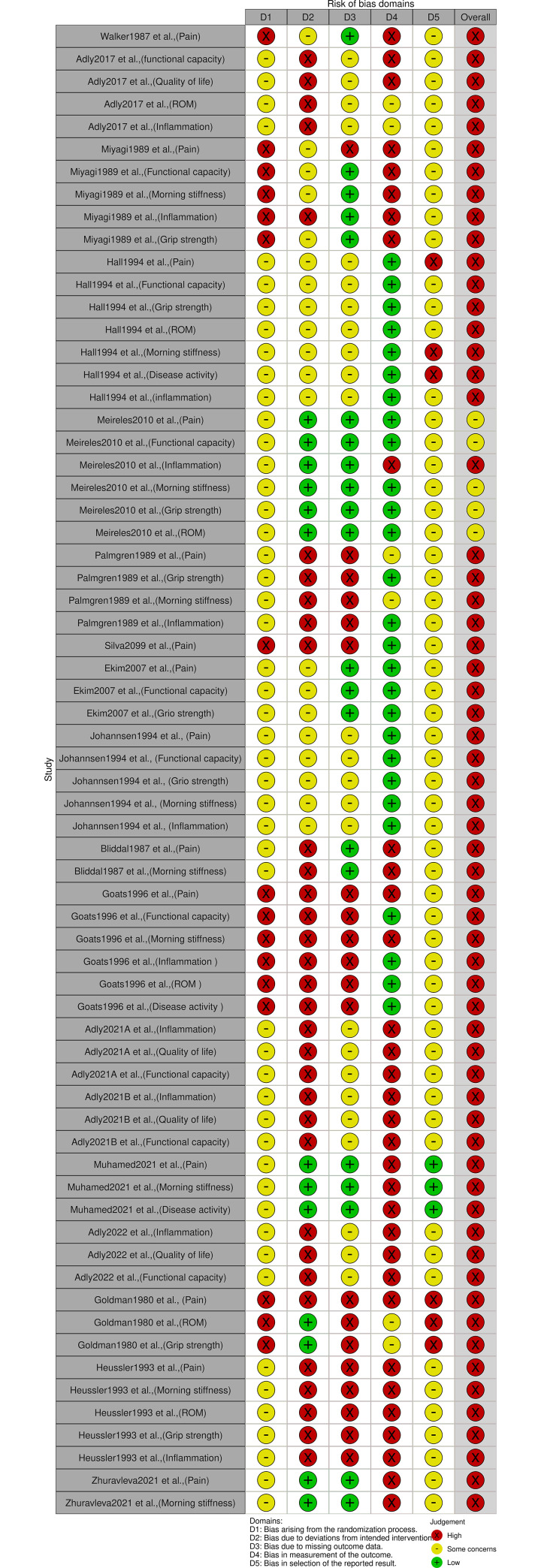
Risk of bias in included studies.

Among the 18 included studies, 17 had an overall high risk of bias and one study [30] had “some concerns” on the overall risk of bias. The judgments for each domain of each risk of bias outcome are detailed in Fig 2. All results of the verification certainty assessment can be found in S4 Table.

### Effects of interventions

#### Comparison: Infrared versus sham

**Fig 3:**
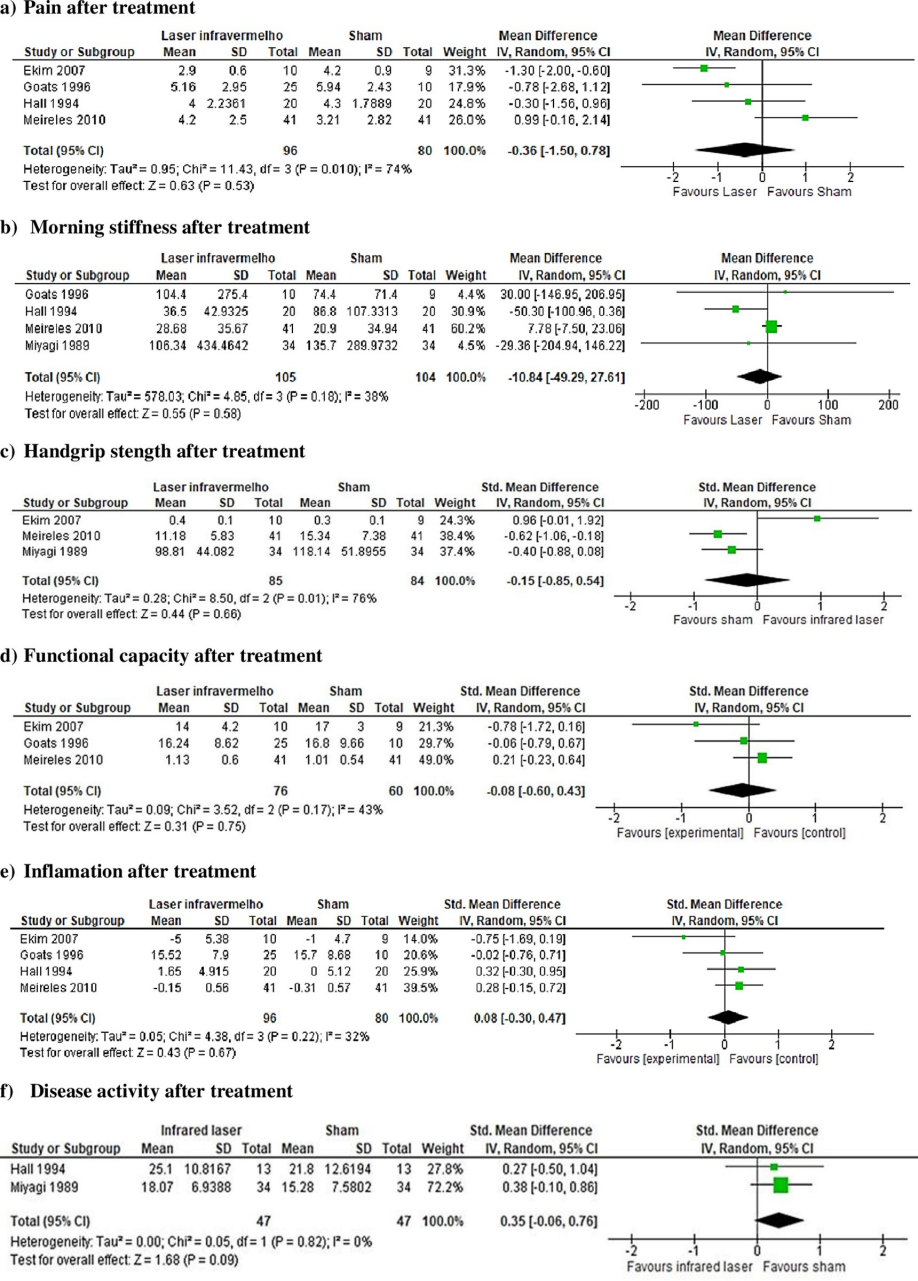
Meta-analysis.

*Pain*. Four studies [24, 25, 27, 30] used a Visual Analogue Scale (VAS) to assess pain. Pooled data from these 4 RCTs, with a total of 176 participants, suggest there may be small or no differences in pain between using laser infrared and sham after 4 to 8 weeks (MD -0.36; 95% CI -1.50 to 0.78; low certainty of evidence) (Fig 3A).

In addition to these studies, Silva et al. [33] evaluated pain with the Br-MPQ Pain Questionnaire. In this study, there was less pain in the sham group (80%) and a result of greater pain in the irradiated group (40%). Johannsen et al. [29] assessed pain using the 0–12 score range instrument and found a reduction on pain score in the infrared laser group, but when data were corrected for disease variation, the effect disappeared. Goldman et al. [26] also reported a significant difference favoring infrared laser group for pain but did not provide further information for analysis. The results of the 3 studies were not pooled with the other 4 RCTs because they were reported in ways that did not allow pooling.

*Morning stiffness*. Four studies evaluated the duration of morning stiffness [25, 27, 30, 31]. To morning stiffness outcome, data from four RCTs were pooled to meta-analysis with a total of 209 individuals. The meta-analysis results showed that there is low certainty of evidence for small or no difference in morning stiffness between using laser infrared compared to sham after 1 to 2 months (MD = - 10.84; 95% CI –49.29 to 27.61) (Fig 3B). Heussler et al. [28] also evaluated the duration of morning stiffness and did not find a significant difference between groups, but did not provide further information for analysis.

*Handgrip strength*. Three studies [24, 30, 31] evaluated handgrip strength and the pooled data from these trials, with a total of 169 participants, suggest there may be small or no difference in handgrip strength between using laser infrared and sham (SMD -0.15; 95% CI -0.85 to 0.54; low certainty of evidence) (Fig 3C). Heussler et al. [28] and Johannsen et al. [29] reported they did not find a significant difference between groups. Goldman et al. [26] and Palmgren et al. [32] reported a significant difference favoring infrared laser group for grip strength. However, these studies did not provide further information for analysis. Hall et al. [27] presented data only in graphs without further information for analysis.

*Functional capacity*. Two studies [25, 30] evaluated functional capacity using the Health Assessment Questionnaire (HAQ) and one [24] using a Functional Scale with 8 items. Pooled data from these 3 RCTs, with a total of 136 participants, suggest there may be small or no difference in functional capacity between using laser infrared and sham (SMD -0.08; 95% CI -0.60 to 0.43; low quality of evidence) (Fig 3D). Hall et al. [27] evaluated functional capacity but presented data only in graphs without further information for analysis.

*Inflammation*. Two studies [25, 31] evaluated c-reactive protein (CRP) (in mg/dl) through blood collection. Pooled data from two studies, with 103 participants, suggest there may be small or no difference between using infrared laser or sham (MD = 0.18; 95% CI ((-0.51 to 0.87) (Fig 3E). Meireles et al. [30], using a Likert scale, (1—no inflammation; 2- mild inflammation; 3-moderate inflammation; 4 strong inflammation; or 5—very strong inflammation), encountered a statistically significant difference (p = 0.012) in favor of the group that received infrared laser. Johannsen et al. [29] and Meireles et al. [30] reported they did not find a significant difference between groups and did not provide enough data for further analysis. Hall [27] presented data only in graphs without further information for analysis.

*Range of motion (ROM)*. Meireles et al. [30] analyzed proximal interphalangeal (PIP) joints using a goniometry, results of this study with 82 participants found a small difference in ROM after 2 months (p = 0.021 favoring control group). Ekim et al. [24] found no significant difference observed between active or placebo groups in ROM at the knee. Goldman et al. [26] and Heussler [28] reported no significant difference between groups for PIP range of motion. Hall et al. [27] presented data only in graphs without further information for analysis. These results suggest there may be small or no difference between laser infrared and sham on ROM (low certainty of evidence).

*Disease activity*. Hall et al. [27] used the Richie index and Miyagi et al. [31] used Lansbury. Pooled data from these studies with a total of 94 participants suggested that there may be small or no difference in disease activity between using infrared laser and sham (SMD 0.35; 95% CI –0.06 to 0.76; low quality of evidence) (Fig 3F).

*Adverse events*. Johannsen et al. [29] reported adverse events related to interventions. In this study, two patients receiving infrared laser treatment had increased disease activity, requiring steroid treatment. These two patients were withdrawn from the study. Two other patients, one from each treatment group, complained of a burning sensation in the treated joints, but completed the study.

Ekim et al. [24] and Miyagi et al. [31] reported that no systemic or local side effects were reported during or after the treatment period. Heussler et al. [28] reported no significant difference between groups for side effects. Johannsen et al. [29] reported no side effects. Due to the few events and risk of bias in studies, the evidence is very uncertain about adverse effects of infrared laser compared to sham (very low certainty of evidence).

#### Comparison: Red laser versus sham

*Pain*. Two studies, Walker et al. [35] and Bliddal et al. [34], evaluated pain outcome after red laser. These studies were not sufficiently homogeneous to be pooled. Walker et al. [35] observed no difference between groups for the pain outcome. According to Bliddal et al. [34], red laser treatment is better than placebo in terms of pain relief. The quality of evidence for this outcome is very low.

*Morning stiffness*. Bliddal et al. [34] evaluated morning stiffness after 10 weeks of red laser. In this study, it was not possible to detect differences between red laser and sham treatment regarding relief of morning stiffness. The quality of evidence for this outcome is very low.

*Adverse events*. For Bliddal et al. [31], adverse effects were observed in 3 patients, who complained of a burning sensation in the irradiated joints—all in the laser-treated group. In these cases, the sensation disappeared within a few hours and none of the patients withdrew from the study. The quality of evidence for this outcome is very low.

#### Comparison: Infrared laser versus laser acupuncture

*Functional capacity*. The results of Adly et al. [38] with 40 participants suggest the evidence is very uncertain about the effects of infrared laser versus laser acupuncture, as assessed by the HAQ (MD 0.01; 95% CI –0.23 to 0.25; very low quality of evidence).

*Quality of life*. The evidence is also very uncertain about the effects of infrared laser versus laser acupuncture in the quality of life using The Rheumatoid Arthritis Quality of Life Questionnaire (RAQoL), after 4 weeks (MD 4.05; 95% CI 0.48 to 7.62; very low quality of evidence).

*Inflammation*. We are uncertain about the effects of infrared laser versus laser acupuncture in inflammation as assessed with interleukin 6 (MD 36.03; 95% CI -0.72 to 72.79; very low quality of evidence).

#### Comparison: Laser acupuncture versus reflexology

*Functional capacity*. The results of Adly [37] with 30 participants suggest the evidence is very uncertain about the effects of laser acupuncture versus reflexology on functional capacity after 4 weeks, as assessed by the HAQ (MD 32.49; 95% CI 28.54 to 36.44; very low quality of evidence).

*Quality of life*. The evidence is also very uncertain about the effects of laser acupuncture versus reflexology in the quality of life using The Rheumatoid Arthritis Quality of Life Questionnaire (RAQoL), after 4 weeks (MD -4.05; 95% CI -9.18 to 1.08; very low quality of evidence).

*Inflammation*. We are uncertain about the effects of laser acupuncture versus reflexology in inflammation as assessed with interleukin 6 (MD 27.7; 95% CI -70.52 to 15.12; very low quality of evidence).

*Range of motion*. There is also very low quality of evidence on the effects of laser acupuncture versus reflexology in the range of motion, including evaluations of plantar flexion and dorsiflexion, wrist flexion and extension, and ulnar and radial deviation, although all differences were favorable to the group receiving laser acupuncture, except for ulnar deviation, where no significant difference between groups was found (Table 1).

#### Comparison: Laser acupuncture + teletherapy + methotrexate versus teletherapy + methotrexate

*Functional capacity*. The results of Adly et al. [38] with 60 participants suggest the evidence is very uncertain about the effects of laser acupuncture + teletherapy + methotrexate versus teletherapy + methotrexate, as assessed by the HAQ. In this study, the authors reported no significant difference between groups, without further data for analysis; very low quality of evidence.

*Quality of life*. We are uncertain about the effects of laser acupuncture versus reflexology in the quality of life using The Rheumatoid Arthritis Quality of Life Questionnaire (RAQoL). The authors found a significant difference between groups favoring laser acupuncture, with a mean difference of –4.533, without further data for analysis; very low quality of evidence.

*Inflammation*. We are uncertain about the effects of laser acupuncture versus reflexology in inflammation as assessed with interleukin 6. The authors found a significant difference between groups favoring laser acupuncture assessed using CRP and IL-6, with a mean difference of –34.68 and –41, respectively, without further data for analysis; very low quality of evidence.

#### Comparison: Laser acupuncture + teletherapy versus teletherapy

*Functional capacity*. The results of Adly et al. [39] with 60 participants suggest the evidence is very uncertain about the effects of laser acupuncture + teletherapy versus teletherapy on functional capacity, as assessed by the HAQ (MD 0.00–0.24 to 0.24; very low quality of evidence).

*Quality of life*. The evidence is also very uncertain about the effects of laser acupuncture + teletherapy versus teletherapy in the quality of life using The Rheumatoid Arthritis Quality of Life Questionnaire (RAQoL), after 4 weeks (MD –4.47; 95% CI -8.20 to –0.74; very low quality of evidence).

*Inflammation*. We are uncertain about the effects of laser acupuncture + teletherapy versus teletherapy in inflammation as assessed with interleukin 6 (MD –35.57; 95% CI –37.63 to –33.5, and MD –31.17; 95% CI –63.1 to 0.76; very low quality of evidence).

#### Comparison: Infrared laser + red laser versus sham laser + naproxen

*Pain*. The results of Al-Saraj et al. [40] with 24 participants suggest the evidence is very uncertain about the effects of infrared laser + red laser versus sham laser + naproxen on pain (MD -9.05; 95% CI -17.52 to –0.58; very low quality of evidence).

*Morning stiffness*. The results suggest the evidence is very uncertain about the effects of infrared laser + red laser versus sham laser + naproxen on morning stiffness (MD -7.4; 95% CI -13.4 to –1.4; very low quality of evidence).

*Inflammation*. We are uncertain about the effects of infrared laser + red laser versus sham laser + naproxen in inflammation as assessed with erythrocyte sedimentation rate (ESR) (MD –4.1; 95% CI -10.26 to 2.06; very low quality of evidence).

*Disease activity*. The evidence is also very uncertain about the effects of infrared laser + red laser versus sham laser + naproxen in the quality of life using Disease Activity Score 28 (DAS-28) (MD -0.49; 95% CI -1.00 to 0.02; very low quality of evidence).

*Adverse events*. The evidence is also very uncertain about the adverse effects of infrared laser + red laser versus sham laser + naproxen (RR 0.14; 95% CI 0.01 to 2.50; very low quality of evidence).

#### Comparison: Infrared laser + red laser versus naproxen

*Pain*. The results of Al-Saraj et al. [40] with 22 participants suggest the evidence is very uncertain about the effects of infrared laser + red laser versus sham laser + naproxen on pain (MD -7.3; 95% CI -17.33 to 2.73; very low quality of evidence).

*Morning stiffness*. The results of this study [40] with 22 participants suggest the evidence is very uncertain about the effects of infrared laser + red laser versus sham laser + naproxen on morning stiffness (MD -9.50; 95% CI -15.10 to –3.90; very low quality of evidence).

*Inflammation*. We are uncertain about the effects of infrared laser + red laser versus sham laser + naproxen in inflammation as assessed with ESR (MD –4.50; 95% CI –68.48 to 59.48; very low quality of evidence).

*Disease activity*. The evidence is also very uncertain about the effects of infrared laser + red laser versus sham laser + naproxen in the quality of life using Disease Activity Score 28 (DAS-28) (MD -0.22; 95% CI –0.67 to 0.23; very low quality of evidence).

*Adverse events*. The evidence is also very uncertain about the adverse effects of infrared laser + red laser versus naproxen (RR 0.17; 95% CI 0.01 to 3.16; very low quality of evidence).

#### Comparison: Infrared laser + methotrexate + non-steroidal anti-inflammatory drugs (NSAIDs) on demand versus methotrexate + NSAIDs on demand

*Pain*. Zhuravleva et al. [41] compared Infrared laser + methotrexate + NSAIDS versus methotrexate + NSAIDS using a Visual Analogue Scale (VAS). This study found a small difference between using infrared laser and control after 6 months of treatment (MD –0.51; IC 95% -0.60 to –0.42; low-certainty of evidence).

*Duration of morning stiffness*. The results of this study [41] suggest participants in the laser group had a shorter duration of morning stiffness compared to control (MD -17.8; IC 95% -19.67 to -15.93; low certainty of evidence).

*Number of patients needing NSAID*. Zhuravleva et al. [41] found that laser led to a decrease in the frequency of the need for NSAIDs compared to control (RR 2.1; IC 95% 1.09 to 4.05; low certainty of evidence).

## Discussion/Conclusion

### Main findings

This systematic review evaluated the current evidence on the effectiveness of LLLT in adult patients with RA. The last systematic review evaluating exclusively adults with RA was published [12] 17 years ago, showing the importance of updating the evidence through this systematic review. Our results show that there is low-quality evidence to suggest that there may be small to no differences between using infrared laser using sham in terms of pain, morning stiffness, grip strength, functional capacity, inflammation, range of motion, disease activity and adverse events. We also found that the evidence is very uncertain about the effects of red laser versus sham in pain, morning stiffness and adverse events and about the effect of laser acupuncture versus reflexology in functional capacity, quality of life and inflammation.

In this systematic review, we included 18 RCTs, of which ten showed there may be no difference between using infrared laser and using sham in any of the outcomes evaluated. Our results are different from the results of Brosseau et al. [12], where LLLT was found to reduce pain and stiffness in a short period when compared to sham. This difference in result may be due to the fact that, in our review, we chose to separate the LLLT with red wavelength from the infrared wavelength, since the infrared LLLT penetrates more into the tissue than does the red laser [9], which Brosseau et al. [12] was unable to do as they included only a few heterogeneous studies. Furthermore, no certainty of evidence assessment was conducted in the systematic review by Brosseau et al. [12].

It is noteworthy that in Brosseau et al. [12], only studies published in French and English were included, which resulted in the inclusion of 5 studies. In this review, the authors reported that despite the positive results found, it was not possible to determine which LLLT parameters are responsible for these effects. Noting that, it is important that future reviews separate the LLLT parameters in the meta-analyses according to wavelength, treatment time, dose and laser application site when possible.

We also found that the evidence is very uncertain about the effects of red laser versus sham in pain, morning stiffness, and adverse events and about the effect of laser acupuncture versus reflexology in functional capacity, quality of life and inflammation. Some studies suggest that LLLT has promising results in controlling joint inflammation [43], reducing pain, tumor necrosis factor alpha (TNF-a) [44] and modulating the inflammatory process [45]. However, to achieve these effects, LLLT depends on specific and important parameters that determine the interaction of laser light with tissue. However, the heterogeneity of the included studies in terms of sample size, treatment time, place of LLLT application and lack of uniformity in the presentation of LLLT parameters make it difficult to interpret the results found and the effectiveness of this resource cannot be proven.

We consider as limitations of this study the inclusion of trials without adequate randomization, studies with small sample data that favor imprecision, resulting in low estimation of effects, and the heterogeneity of laser parameters found. Another limitation of this review would be the estimation of long-term effects that were observed in only two studies. We consider the strengths of this systematic review to be the use of a rigorous methodology, assessment of the risk of bias and the quality of evidence for each outcome, as well as the conduction of broad searches, without publication date or language restrictions.

In conclusion, infrared laser may not be superior to sham in RA patients. There is insufficient evidence to support or refute the effectiveness of red laser, laser acupuncture and reflexology for treating patients with RA. Further studies with more rigorous scientific methodology and larger sample size are needed to monitor the effects of LLT in patients with RA in the long term.

## Supporting information

S1 Table Search strategy for included studies. (DOCX)

S2 Table Preferred Reporting Items for Systematic Reviews and Meta-Analysis (PRISMA). (DOCX)

S3 Table Studies awaiting classification. (DOCX)

S4 Table Grading of Recommendations Assessment, Development and Evaluation (GRADE). (DOCX)
