# Supplementary material for: Effects of low-level laser therapy in adults with rheumatoid arthritis: A systematic review and meta-analysis of controlled trials
Source: PLoS One. 2023 Sep 8;18(9):e0291345. doi: 10.1371/journal.pone.0291345 (PMC10490856; doi:10.1371/journal.pone.0291345)
Supplement: S1 Table — (DOCX) [file pone.0291345.s001.docx]

S1 Table. Search strategy for included studies.

| MEDLINE via PubMed | ((((("Arthritis, Rheumatoid"[Mesh]) OR (rheumatoid arthritis) OR (degenerative arthritis) OR  rheumatism OR ("Caplan Syndrome"[Mesh]) OR (caplan disease) OR (caplan's syndrome) OR  ("Felty Syndrome"[Mesh])))) AND ((("Lasers"[Mesh] OR laser OR ("Laser Therapy"[Mesh])  OR ("Low-Level Light Therapy"[Mesh]) OR LLLT OR (light therapies, low-level) OR (light  therapy, low-level) OR (low level light therapy) OR (low-level light therapies) OR (therapies,  low-level light) OR (therapy, low-level light) OR photobiomodulation OR ("Infrared  Rays"[Mesh]) OR infrared) OR monochromatic OR ("Ultraviolet Rays"[Mesh]) OR  ultraviolet))) AND (((clinical[Title/Abstract] AND trial[Title/Abstract]) OR clinical trials as  topic[MeSH Terms] OR clinical trial[Publication Type] OR random*[Title/Abstract] OR  random allocation[MeSH Terms] OR therapeutic use[MeSH Subheading])) | 668 |
| --- | --- | --- |
| Cochrane Central Register of Controlled Trials (CENTRAL) | #1 MeSH descriptor: [Arthritis, Rheumatoid] explode all trees 5987  #2 MeSH descriptor: [Caplan Syndrome] explode all trees 0  #3 MeSH descriptor: [Felty Syndrome] explode all trees 0  53  #4 (rheumatoid arthritis) OR (degenerative arthritis) OR rheumatism OR (caplan disease)  OR (caplan's syndrome) 18615  #5 #1 OR #2 OR #3 OR #4 18868  #6 MeSH descriptor: [Lasers] explode all trees 2061  #7 MeSH descriptor: [Laser Therapy] explode all trees 3983  #8 MeSH descriptor: [Low-Level Light Therapy] explode all trees 906  #9 MeSH descriptor: [Infrared Rays] explode all trees 214  #10 MeSH descriptor: [Ultraviolet Rays] explode all trees 657  #11 laser OR (light therapies, low-level) OR (light therapy, low-level) OR (low level light  therapy) OR (low-level light therapies) OR (therapies, low-level light) OR (therapy, low-level  light) OR photobiomodulation OR infrared OR monochromatic OR ultraviolet 26928  #12 #6 or #7 or #8 or #9 Or #10 or #11 27073  #13 #5 AnD #12 292  Trials 138 | 138 |
| Embase | #1 - 'rheumatoid arthritis'/exp OR 'arthritis, rheumatoid' OR 'degenerative arthritis' OR 'Felty  syndrome'/exp OR ‘syndrome felty’ OR 'rheumatic arthritis' OR rheumatism OR 'Caplan  disease' OR 'Caplan syndrome'  #2 - 'laser'/exp OR 'low level laser therapy'/exp OR laser OR 'low level light therapy' OR 'lowlevel  light therapy' OR 'photobiomodulation'/exp OR 'infrared radiation'/exp OR infrared OR  'monochromatic light'/exp OR 'ultraviolet radiation'/exp OR ultraviolet  #3 - #1 AND #2  #4 - 'crossover procedure':de OR 'double-blind procedure':de OR 'randomized controlled  trial':de OR 'single-blind procedure':de OR random*:de,ab,ti OR factorial*:de,ab,ti OR  crossover*:de,ab,ti OR ((cross NEXT/1 over*):de,ab,ti) OR placebo*:de,ab,ti OR ((doubl*  NEAR/1 blind*):de,ab,ti) OR ((singl* NEAR/1 blind*):de,ab,ti) OR assign*:de,ab,ti OR  allocat*:de,ab,ti OR volunteer*:de,ab,ti  #5 - #3 AND #4 | 281 |
| Latin American and Caribbean Health Sciences Literature (LILACS) via Portal BVS = LILACS, IBECS, CUMED | MH:"Artrite Reumatoide" OR (Arthritis, Rheumatoid) OR (Artritis Reumatoide) OR (Artrite  Reumatoide) OR MH:C05.550.114.154$ OR MH:C05.799.114$ OR MH:C17.300.775.099$  OR MH:C20.111.199$ OR MH:"Síndrome de Caplan" OR (Caplan Syndrome) OR  MH:C05.550.114.154.219$ OR MH:C05.799.114.219$ OR MH:C08.381.483.581.300$ OR  MH:C08.381.520.702.300$ OR MH:C17.300.775.099.219$ OR MH:C24.800.340$ OR  MH:"Síndrome de Felty" OR (Felty Syndrome) OR (Síndrome de Felty) OR  MH:C05.550.114.154.389$ OR MH:C05.799.114.389$ OR MH:C17.300.775.099.389$ OR  MH:C20.111.199.389$  MH:Lasers OR Lasers OR (Rayos Láser) OR Laser OR Maser OR Masers OR  MH:E07.632.490$ OR MH:E07.710.520$ OR MH:SP4.011.087.698.384.075.166.027$ OR  MH:VS2.006.002.009$ OR MH:"Terapia a Laser" OR (Terapia por Láser) OR (Laser Therapy)  OR MH:E02.594$ OR MH:E04.014.520$ OR MH:"Terapia com Luz de Baixa Intensidade"  OR (Terapia por Luz de Baja Intensidad) OR (Low-Level Light Therapy) OR LLLT OR  MH:E02.594.540$ OR MH:E02.774.500$ OR Fotobiomodulação OR MH:"Raios  Infravermelhos" OR (Rayos Infrarrojos) OR (Infrared Rays) OR (Ondas de Calor) OR  MH:G01.358.500.505.650.552$ OR MH:G01.590.540.552$ OR MH:G01.750.250.650.552$  OR MH:G01.750.770.578.552$ OR MH:G16.500.275.063.725.525.400$ OR  MH:G16.500.750.775.525.400$ OR MH:N06.230.300.100.725.525.400$ OR  MH:SP4.011.087.698.384.075.176.052$ OR monocromatica OR MH:"Raios Ultravioleta" OR  (Rayos Ultravioleta) OR (Ultraviolet Rays) OR ultravioleta OR  MH:G01.358.500.505.650.891$ OR MH:G01.590.540.891$ OR MH:G01.750.250.650.891$  OR MH:G01.750.750.659$ OR MH:G01.750.770.578.891$ OR  MH:G16.500.275.063.725.525.600$ OR MH:G16.500.750.775.525.600$ OR  MH:N06.230.300.100.725.525.600$ OR MH:SP4.011.087.698.384.075.166.032$ OR  MH:SP4.021.202.133.789$ | 15 |
| PEDro | Abstract & Title: "rheumatoid arthritis" laser  Method: Clinical trial | 16 |
| SciELO | Laser AND “rheumatoid arthritis | 3 |
