# Supplementary material for: Effects of low-level laser therapy in adults with rheumatoid arthritis: A systematic review and meta-analysis of controlled trials
Source: PLoS One. 2023 Sep 8;18(9):e0291345. doi: 10.1371/journal.pone.0291345 (PMC10490856; doi:10.1371/journal.pone.0291345)
Supplement: S3 Table — (DOCX) [file pone.0291345.s003.docx]

**S4 Table**. Grading of Recommendations Assessment, Development and Evaluation (GRADE).

| **Infrared laser compared to sham for RA** | | | | | | | | | | | |
| --- | --- | --- | --- | --- | --- | --- | --- | --- | --- | --- | --- |
| **Certainty assessment** | | | | | | | **Summary of findings** | | | | |
| **Participants (studies) Follow-up** | **Risk of bias** | **Inconsistency** | **Indirectness** | **Imprecision** | **Publication bias** | **Overall certainty of evidence** | **Study event rates (%)** | | **Relative effect (95% CI)** | **Anticipated absolute effects** | |
|  |  |  |  |  |  |  | **With sham** | **With infrared laser** |  | **Risk with sham** | **Risk difference with infrared laser** |
| **Pain** | | | | | | | | | | | |
| 176 (4 RCTs) | serious^a^ | not serious | not serious | serious^b^ | none | ⨁⨁◯◯ Low | 80 | 96 | - | The mean pain was **0** SD | MD **0.36 SD lower** (1.5 lower to 0.78 higher) |
| **Morning stiffness** | | | | | | | | | | | |
| 209 (4 RCTs) | serious^a^ | not serious | not serious | serious^b^ | none | ⨁⨁◯◯ Low | 104 | 105 | - | The mean morning stiffness was **0** | MD **10.84 lower** (49.29 lower to 27.61 higher) |
| **Grip strength** | | | | | | | | | | | |
| 169 (3 RCTs) | serious^a^ | not serious | not serious | serious^b^ | none | ⨁⨁◯◯ Low | 84 | 85 | - | - | SMD **0.15 SD lower** (0.85 lower to 0.54 higher) |
| **Functional Capacity** | | | | | | | | | | | |
| 136 (3 RCTs) | serious^a^ | not serious | not serious | serious^b^ | none | ⨁⨁◯◯ Low | 60 | 76 | - | - | SMD **0.08 SD lower** (0.6 lower to 0.43 higher) |
| **Inflammation (C-reactive protein)** | | | | | | | | | | | |
| 103 (3 RCTs) | serious^a^ | not serious | not serious | serious^b^ | none | ⨁⨁◯◯ Low | 44 | 59 | - | The mean inflammation (C-reactive protein) was **0** | MD **0.18 higher** (0.51 lower to 0.87 higher) |
| **Range of motion** | | | | | | | | | | | |
| 237 (5 RCTs) | serious^a^ | not serious | not serious | serious^b^ | none | ⨁⨁◯◯ Low | Results of Meireles 2010 with a total of 82 participants found a small difference in ROM after 2 months (p = 0.021 favoring control group). In Goats et al., 1996 (35 participants) , there were no significant differences observed between the active or placebo groups for the range of movement at the knee. Goldman et al., 1980 (30 participants) and Heussler et al., 1993 (25 participants; 50hands) reported no significant difference between groups for PIP range of motion. Hall et al., 1994 (40 participants) presented data only in graphs without further information for analysis | | | | |

**CI:** confidence interval; **MD:** mean difference; **SMD:** standardised mean difference

**Explanations**

a. We downgraded one level due to serious risk of bias

b. We downgraded one level due to small sample size

| **Red laser compared to sham for RA** | | | | | | | | | | |
| --- | --- | --- | --- | --- | --- | --- | --- | --- | --- | --- |
| **Certainty assessment** | | | | | | | **Summary of findings** | | | |
| **Participants (studies) Follow-up** | **Risk of bias** | **Inconsistency** | **Indirectness** | **Imprecision** | **Publication bias** | **Overall certainty of evidence** | **Study event rates (%)** | |  |  |
|  |  |  |  |  |  |  | **With sham** | **With red laser** |  |  |
| **Pain** | | | | | | | | | | |
| 89 (2 RCTs) | serious^a^ | serious^b^ | not serious | serious^c^ | none | ⨁◯◯◯ Very low | Walker 1987 (72 participans) reported no differences were observed between groups for the pain outcome. For Bliddal 1987 (17 participants) red laser treatment is better than placebo in terms of pain relief. | | | |
| **Morning stiffness** | | | | | | | | | | |
| 17 (1 RCT) | serious^a^ | not serious | not serious | very serious^d^ | none | ⨁◯◯◯ Very low | One study (Bliddal 1987) (17 participants) evaluated morning stiffness after 10 weeks of red laser. In Bliddal 1987, it was not possible to detect differences between red laser and sham treatment with respect to relief of morning stiffness. | | | |
| **Adverse events** | | | | | | | | | | |
| 17 (1 RCT) | serious^a^ | not serious | not serious | very serious^d^ | none | ⨁◯◯◯ Very low | Adverse events reported by Bliddal (1987) (17 participants). In this study, adverse effects were observed in 3 patients, who complained of a burning sensation in the irradiated joints - all in the laser-treated group. In these cases, the sensation disappeared within a few hours and none of the patients withdrew from the study. | | | |

**CI:** confidence interval

**Explanations**

a. We downgraded one level due to serious risk of bias

b. We downgraded one level due to serious inconsistency

c. We downgraded one level due to small sample size

d. We downgraded two levels due to very serious imprecision (very small sample size or small number of events)

| **Infrared laser compared to laser acupuncture for RA** | | | | | | | | | | | |
| --- | --- | --- | --- | --- | --- | --- | --- | --- | --- | --- | --- |
| **Certainty assessment** | | | | | | | **Summary of findings** | | | | |
| **Participants (studies) Follow-up** | **Risk of bias** | **Inconsistency** | **Indirectness** | **Imprecision** | **Publication bias** | **Overall certainty of evidence** | **Study event rates (%)** | | **Relative effect (95% CI)** | **Anticipated absolute effects** | |
|  |  |  |  |  |  |  | **With laser acupuncture** | **With infrared laser** |  | **Risk with laser acupuncture** | **Risk difference with infrared laser** |
| **Functional capacity** | | | | | | | | | | | |
| 40 (1 RCT) | serious^a^ | not serious | not serious | very serious^b^ | none | ⨁◯◯◯ Very low | 20 | 20 | - | The mean functional capacity was **0** | MD **0.01 higher** (0.23 lower to 0.25 higher) |
| **Quality of life** | | | | | | | | | | | |
| 40 (1 RCT) | serious^a^ | not serious | not serious | very serious^b^ | none | ⨁◯◯◯ Very low | 20 | 20 | - | The mean quality of life was **0** | MD **4.05 higher** (0.48 higher to 7.62 higher) |
| **Inflammation** | | | | | | | | | | | |
| 40 (1 RCT) | serious^a^ | not serious | not serious | very serious^b^ | none | ⨁◯◯◯ Very low | 20 | 20 | - | The mean inflammation was **0** | MD **36.03 higher** (0.72 lower to 72.79 higher) |

**CI:** confidence interval; **MD:** mean difference

**Explanations**

a. We downgraded one level due to serious risk of bias

b. We downgraded two levels due to very serious imprecision (very small sample size and/or large confidence interval)

| **Laser acupuncture compared to reflexology for RA** | | | | | | | | | | | |
| --- | --- | --- | --- | --- | --- | --- | --- | --- | --- | --- | --- |
| **Certainty assessment** | | | | | | | **Summary of findings** | | | | |
| **Participants (studies) Follow-up** | **Risk of bias** | **Inconsistency** | **Indirectness** | **Imprecision** | **Publication bias** | **Overall certainty of evidence** | **Study event rates (%)** | | **Relative effect (95% CI)** | **Anticipated absolute effects** | |
|  |  |  |  |  |  |  | **With reflexology** | **With laser acupuncture** |  | **Risk with reflexology** | **Risk difference with laser acupuncture** |
| **Functional capacity** | | | | | | | | | | | |
| 30 (1 RCT) | serious^a^ | not serious | not serious | very serious^b^ | none | ⨁◯◯◯ Very low | 15 | 15 | - | The mean functional capacity was **0** | MD **32.49 higher** (28.54 higher to 36.44 higher) |
| **Quality of life** | | | | | | | | | | | |
| 30 (1 RCT) | serious^a^ | not serious | not serious | very serious^b^ | none | ⨁◯◯◯ Very low | 15 | 15 | - | The mean quality of life was **0** | MD **4.05 lower** (9.18 lower to 1.08 higher) |
| **IL-6** | | | | | | | | | | | |
| 30 (1 RCT) | serious^a^ | not serious | not serious | very serious^b^ | none | ⨁◯◯◯ Very low | 15 | 15 | - | The mean IL-6 was **0** | MD **27.7 lower** (70.52 lower to 15.12 higher) |
| **Range of motion** | | | | | | | | | | | |
| 30 (1 RCT) | serious^a^ | not serious | not serious | very serious^b^ | none | ⨁◯◯◯ Very low | We are uncertain about the effects of laser acupuncture versus reflexology in the range of motion, including evaluations of plantar flexion and dorsiflexion, wrist flexion and extension, and ulnar and radial deviation, although all differences were favorable to the group receiving laser acupuncture, except for ulnar deviation, where no significant difference between groups were found (one study; 30 participants) (Adly et al., 2017) | | | | |

**CI:** confidence interval; **MD:** mean difference

**Explanations**

a. We downgraded one level due to serious risk of bias

b. We downgraded two levels due to very serious imprecision (very small sample size and/or large confidence interval)

| **Laser acupuncture + teletherapy + methotrexate compared to teletherapy + methotrexate for RA** | | | | | | | | | | | |
| --- | --- | --- | --- | --- | --- | --- | --- | --- | --- | --- | --- |
| **Certainty assessment** | | | | | | | **Summary of findings** | | | | |
| **Participants (studies) Follow-up** | **Risk of bias** | **Inconsistency** | **Indirectness** | **Imprecision** | **Publication bias** | **Overall certainty of evidence** | **Study event rates (%)** | | **Relative effect (95% CI)** | **Anticipated absolute effects** | |
|  |  |  |  |  |  |  | **With teletherapy + methotrexate** | **With Laser acupuncture + teletherapy + methotrexate** |  | **Risk with teletherapy + methotrexate** | **Risk difference with Laser acupuncture + teletherapy + methotrexate** |
| **Functional capacity** | | | | | | | | | | | |
| 60 (1 RCT) | serious^a^ | not serious | not serious | very serious^b^ | none | ⨁◯◯◯ Very low | . Adly, 2022, reported no significant difference between groups as assessed with HAQ, without further data for analysis | | | | |
| **Quality of life** | | | | | | | | | | | |
| 60 (1 RCT) | serious^a^ | not serious | not serious | very serious^b^ | none | ⨁◯◯◯ Very low | Adly, 2022, reported a significant difference between groups favoring laser acupuncture, with a mean difference of –4.533, without further data for analysis | | | | |
| **Inflammation** | | | | | | | | | | | |
| 60 (1 RCT) | serious^a^ | not serious | not serious | very serious^b^ | none | ⨁◯◯◯ Very low | Adly, 2022, reported a significant difference between groups favoring laser acupuncture assessed using CRP and IL-6, with a mean difference of –34.68 and –41, respectively, without further data for analysis. | | | | |

**CI:** confidence interval; **MD:** mean difference

**Explanations**

a. We downgraded one level due to serious risk of bias

b. We downgraded two levels due to very serious imprecision (small sample size and/or large confidence interval)

| **Laser acupuncture + teletherapy compared to teletherapy for RA** | | | | | | | | | | | |
| --- | --- | --- | --- | --- | --- | --- | --- | --- | --- | --- | --- |
| **Certainty assessment** | | | | | | | **Summary of findings** | | | | |
| **Participants (studies) Follow-up** | **Risk of bias** | **Inconsistency** | **Indirectness** | **Imprecision** | **Publication bias** | **Overall certainty of evidence** | **Study event rates (%)** | | **Relative effect (95% CI)** | **Anticipated absolute effects** | |
|  |  |  |  |  |  |  | **With teletherapy** | **With laser acupuncture + teletherapy** |  | **Risk with teletherapy** | **Risk difference with laser acupuncture + teletherapy** |
| **Functional Capacity** | | | | | | | | | | | |
| 60 (1 RCT) | serious^a^ | not serious | not serious | very serious^b^ | none | ⨁◯◯◯ Very low | 30 | 30 | - |  | MD **0**  (0.24 lower to 0.24 higher) |
| **Quality of life** | | | | | | | | | | | |
| 60 (1 RCT) | serious^a^ | not serious | not serious | very serious^b^ | none | ⨁◯◯◯ Very low | 30 | 30 | - |  | MD **4.47 lower** (8.2 lower to 0.74 lower) |
| **Inflammation (c-reactive protein)** | | | | | | | | | | | |
| 60 (1 RCT) | serious^a^ | not serious | not serious | very serious^b^ | none | ⨁◯◯◯ Very low | 30 | 30 | - |  | MD **35.59 lower** (37.29 lower to 33.89 lower) |
| **Inflammation (interleukin-6)** | | | | | | | | | | | |
| 60 (1 RCT) | serious^a^ | not serious | not serious | very serious^b^ | none | ⨁◯◯◯ Very low | 30 | 30 | - |  | MD **31.17 lower** (59.31 lower to 3.03 lower) |

**CI:** confidence interval; **MD:** mean difference

**Explanations**

a. We downgraded one level due to serious risk of bias

b. We downgraded two level due to very serious imprecision (small sample size and/or large confidence interval)

| **Infrared laser + red laser compared to sham laser + naproxen for RA** | | | | | | | | | | | |
| --- | --- | --- | --- | --- | --- | --- | --- | --- | --- | --- | --- |
| **Certainty assessment** | | | | | | | **Summary of findings** | | | | |
| **Participants (studies) Follow-up** | **Risk of bias** | **Inconsistency** | **Indirectness** | **Imprecision** | **Publication bias** | **Overall certainty of evidence** | **Study event rates (%)** | | **Relative effect (95% CI)** | **Anticipated absolute effects** | |
|  |  |  |  |  |  |  | **With sham laser + naproxen** | **With Infrared laser + red laser** |  | **Risk with sham laser + naproxen** | **Risk difference with Infrared laser + red laser** |
| **Pain** | | | | | | | | | | | |
| 24 (1 RCT) | serious^a^ | not serious | not serious | very serious^b^ | none | ⨁◯◯◯ Very low | 12 | 12 | - | The mean pain was **0** | MD **9.05 lower** (17.52 lower to 0.58 lower) |
| **Morning stiffness** | | | | | | | | | | | |
| 24 (1 RCT) | serious^a^ | not serious | not serious | very serious^b^ | none | ⨁◯◯◯ Very low | 12 | 12 | - |  | MD **7.4 lower** (13.4 lower to 1.4 lower) |
| **Inflammation (erythrocyte sedimentation rate)** | | | | | | | | | | | |
| 24 (1 RCT) | serious^a^ | not serious | not serious | very serious^b^ | none | ⨁◯◯◯ Very low | 12 | 12 | - |  | MD **4.1 lower** (10.26 lower to 2.06 higher) |
| **Disease Activity (DAS-28)** | | | | | | | | | | | |
| 24 (1 RCT) | serious^a^ | not serious | not serious | very serious^b^ | none | ⨁◯◯◯ Very low | 12 | 12 | - |  | MD **0.49 lower** (1 lower to 0.02 higher) |
| **Adverse events** | | | | | | | | | | | |
| 24 (1 RCT) | serious^a^ | not serious | not serious | very serious^c^ | none | ⨁◯◯◯ Very low | 3/12 (25.0%) | 0/12 (0.0%) | **RR 0.14** (0.01 to 2.50) | 250 per 1,000 | **215 fewer per 1,000** (from 248 fewer to 375 more) |

**CI:** confidence interval; **MD:** mean difference; **RR:** risk ratio

**Explanations**

a. We downgraded one level due to serious risk of bias

b. We downgraded two levels due to very serious imprecision (small sample size and/or large confidence interval)

c. We downgraded two levels due to very serious imprecision (few events)

| **Infrared laser + red laser compared to naproxen for RA** | | | | | | | | | | | |
| --- | --- | --- | --- | --- | --- | --- | --- | --- | --- | --- | --- |
| **Certainty assessment** | | | | | | | **Summary of findings** | | | | |
| **Participants (studies) Follow-up** | **Risk of bias** | **Inconsistency** | **Indirectness** | **Imprecision** | **Publication bias** | **Overall certainty of evidence** | **Study event rates (%)** | | **Relative effect (95% CI)** | **Anticipated absolute effects** | |
|  |  |  |  |  |  |  | **With naproxen** | **With Infrared laser + red laser** |  | **Risk with naproxen** | **Risk difference with Infrared laser + red laser** |
| **Pain** | | | | | | | | | | | |
| 22 (1 RCT) | serious^a^ | not serious | not serious | very serious^b^ | none | ⨁◯◯◯ Very low | 10 | 12 | - |  | MD **7.3 lower** (17.33 lower to 2.73 higher) |
| **Morning stiffness** | | | | | | | | | | | |
| 22 (1 RCT) | serious^a^ | not serious | not serious | very serious^b^ | none | ⨁◯◯◯ Very low | 10 | 12 | - |  | MD **9.5 lower** (15.1 lower to 3.9 lower) |
| **Inflammation (erythrocyte sedimentation rate)** | | | | | | | | | | | |
| 22 (1 RCT) | serious^a^ | not serious | not serious | very serious^b^ | none | ⨁◯◯◯ Very low | 10 | 12 | - |  | MD **4.5 lower** (68.48 lower to 59.48 higher) |
| **Disease Activity (DAS-28)** | | | | | | | | | | | |
| 22 (1 RCT) | serious^a^ | not serious | not serious | very serious^b^ | none | ⨁◯◯◯ Very low | 10 | 12 | - |  | MD **0.22 lower** (0.67 lower to 0.23 higher) |
| **Adverse effects** | | | | | | | | | | | |
| 22 (1 RCT) | serious^a^ | not serious | not serious | very serious^c^ | none | ⨁◯◯◯ Very low | 2/10 (20.0%) | 0/12 (0.0%) | **RR 0.17** (0.01 to 3.16) |  | **166 fewer per 1,000** (from 198 fewer to 432 more) |

**CI:** confidence interval; **MD:** mean difference; **RR:** risk ratio

**Explanations**

a. We downgraded one level due to serious risk of bias

b. We downgraded two levels due to very serious imprecision (small sample size and/or large confidence interval)

c. We downgraded two levels due to very serious imprecision (few events and/or large confidence interval)

| **Infrared laser + methotrexate + Non-steroidal anti-inflammatory drugs (NSAIDs) on demand compared to methotrexate + NSAIDs on demand for RA** | | | | | | | | | | | |
| --- | --- | --- | --- | --- | --- | --- | --- | --- | --- | --- | --- |
| **Certainty assessment** | | | | | | | **Summary of findings** | | | | |
| **Participants (studies) Follow-up** | **Risk of bias** | **Inconsistency** | **Indirectness** | **Imprecision** | **Publication bias** | **Overall certainty of evidence** | **Study event rates (%)** | | **Relative effect (95% CI)** | **Anticipated absolute effects** | |
|  |  |  |  |  |  |  | **With methotrexate + NSAIDs on demand** | **With infrared laser + methotrexate + Non-steroidal anti-inflammatory drugs (NSAIDs) on demand** |  | **Risk with methotrexate + NSAIDs on demand** | **Risk difference with infrared laser + methotrexate + Non-steroidal anti-inflammatory drugs (NSAIDs) on demand** |
| **Pain** | | | | | | | | | | | |
| 114 (1 RCT) | very serious^a^ | not serious | not serious | serious^b^ | none | ⨁◯◯◯ Very low | 57 | 57 | - |  | MD **0.51 lower** (0.6 lower to 0.42 lower) |
| **Morning stiffness (duration in min)** | | | | | | | | | | | |
| 114 (1 RCT) | very serious^a^ | not serious | not serious | serious^b^ | none | ⨁◯◯◯ Very low | 57 | 57 | - |  | MD **17.8 lower** (19.67 lower to 15.93 lower) |
| **Number of patients needing non-steroidal anti-inflammatory drugs** | | | | | | | | | | | |
| 114 (1 RCT) | very serious^a^ | not serious | not serious | serious^c^ | none | ⨁◯◯◯ Very low | 10/57 (17.5%) | 21/57 (36.8%) | **RR 2.10** (1.09 to 4.05) | 175 per 1,000 | **193 more per 1,000** (from 16 more to 535 more) |

**CI:** confidence interval; **MD:** mean difference; **RR:** risk ratio

**Explanations**

a. We downgraded two levels due to very serious risk of bias

b. We downgraded one level due to serious imprecision (small sample size)

c. We downgraded two levels due to very serious imprecision (few events and/or large confidence interval)
