# Supplementary material for: Effects of low-level laser therapy in adults with rheumatoid arthritis: A systematic review and meta-analysis of controlled trials
Source: PLoS One. 2023 Sep 8;18(9):e0291345. doi: 10.1371/journal.pone.0291345 (PMC10490856; doi:10.1371/journal.pone.0291345)
Supplement: S4 Table — (DOCX) [file pone.0291345.s004.docx]

**S3 Table.** Studies awaiting classification.

| **Author, year** | **Reason for being awaiting classification** |
| --- | --- |
| 1. Aleksandrova et al., 2000 | Full-text not available. Russian. |
| 2. Beard, 1990 | Abstract not available |
| 3. Bondarenko et al., 1983 | Full-text not available. Russian. |
| 4. Colov et al., 1987 | Abstract not available |
| 5. Iarema et al., 1987 | Full-text not available. Russian. |
| 6. Lysenko et al., 1995 | Full-text not available. Ukrainian |
| 7. Petrov, 2004 | Full-text not available |
| 8. Sidorov et al., 2000 | Full-text not available. Russian |
| 9. Sidorov et al., 1999 | Full-text not available. Russian |
| 10. Soroka et al., 1989 | Full-text not available. Russian |
| 11. Starodubtseva, et al., 2014 | Full-text not available. Russian |
| 12. Starodubtseva, et al., 2015 | Full-text not available. Russian |
| 13. Starodubtseva et al., 2016 | Full-text not available. Russian |
| 14. Tsurko et al.,1983 | Full-text not available. Russian |
| 15. Tupikin et al., 1980 | Full-text not available. Russian |
| 16. Zvereva et al., 1994 | Full-text not available. Russian |
| 17. Zvereva et al., 1996 | Full-text not available. Russian |
|  |  |

**Reference list of studies awaiting classification.**

1. Aleksandrova, O I., G. N. Ponomarenko, I. M. Lukina, (2000) Low-intensity laser radiation in the treatment of patients with rheumatoid arthritis. 0(3), 28-30.
2. Beard, M (1990) Treatment of rheumatoid arthritis with low power laser. Australian journal of physiotherapy. 36(0), 195.
3. Bondarenko, I. P., A. A. Dubinskiĭ, A. K. Pavlichenko, T.F. Shestakova (1983) Use of a helium-neon laser in the treatment of patients with rheumatoid arthritis. Vrachebnoe delo. 0(6), 30-3.
4. Colov, H., N. Palmgren, M. Windelin, M (1987) Convincing clinical improvement of rheumatoid arthritis by soft laser therapy. 7th Meeting ASLMS Abstracts. Laser surgery medicine. 7(0), 77.
5. Iarema, N. Z., P. S. Nazar, L. V. Zoria (1987). Use of immunomodulating and laser therapy in rheumatoid arthritis patients. Vrachebnoe delo. 0(4), 59-61.
6. Lysenko, H. I., I. M. Handzha, L. F. Matiukha, Z. V. Volobuieva (1995) The combined use of diprospan and laser irradiation of the joints in rheumatoid arthritis patients. Likars'ka sprava.0(5), 77-80.
7. Petrov, A. V. (2004) Effect of low intensity helium-neon laser and decimeter electromagnetic irradiation on functional indices of immune cells in patients with rheumatoid arthritis. Likars'ka sprava. 0(2), 30‐35.
8. Sidorov, V. D., D. R. Mamiliaeva, N. A. Derevnina, S. I. U. Reformatskaia, (2000). Kombinirovannaia lazernaia terapiia revmatoidnogo artrita [The combined laser therapy of rheumatoid arthritis]. Voprosy kurortologii, fizioterapii, i lechebnoi fizicheskoi kultury, (2), 13–18.
9. Sidorov V. D., D. R. Mamiliaeva, E. V. Gontar, S. Reformatskaia (1999) Interaurikuliarnaia lazernaia terapiia revmatoidnogo artrita (Interauricular laser therapy of rheumatoid arthritis) Voprosy Kurortologii, Fizioterapii i Lechebnoi Fizicheskoi Kultury. Problems of Health Resorts, Physiotherapy and Exercise Therapy. (3), 35-43.
10. Soroka N. F. (1989) The laser therapy of rheumatoid arthritis. Terapevticheskii arkhiv. 61(12), 124‐127.
11. Starodubtseva, I. A., L. N. Tsvetikova (2014) The pathogenetic substantiation of new therapeutic approach to the treatment of secondary osteoarthritis in patients with rheumatoid arthritis with basis therapy. Advances in gerontology. 27(3), 531-6.
12. Starodubtseva, I. A., L. V. Vasilieva, A. V. Nikitin (2015) Correction of an inflammatory process with an interleukin-1 inhibitor in the combination treatment of secondary osteoarthritis in the presence of comorbid condition. Terapevticheskii arkhiv. 87(12), 41-48.
13. Starodubtseva, I. A., L. V. Vasilieva, A.V. Nikitin (2016) The Analysis of Efficacy of The Application of Interleukin-1 Inhibitor in the Complex Therapy of Secondary Osteoarthritis Taking Into Account the Dynamics of Clinical and Functional Indicators. Vestnik Rossiiskoi akademii meditsinskikh nauk. (2), 141-7.
14. Tsurko V. V. P. la. Mul'diiarov, Ia A. Sigidin (1983) Lazernaia terapiia revmatoidnogo artrita (kliniko-morfologicheskoe issledovanie) [Laser therapy of rheumatoid arthritis (clinico-morphological study)]. Soviet Archives of Internal Medicine. 55(7), 97-102.
15. Tupikin, G. V., A. I. Nesterov, V. P. Gurbanov, T. A. Redina (1980) Effect of laser irradiation on the affected joints in rheumatoid arthritis. Voprosy revmatizma. 0(4), 24-7.
16. Zvereva, K. V., N. D. Gladkova, E. A. Grunina, P. L. Logunov (1994) The choice of the method for intravascular laser therapy in rheumatoid arthritis. Terapevticheskii arkhiv. 66(1), 29-32.
17. Zvereva, K. V., E. A. Grunina (1996) The negative effects of low-intensity laser therapy in rheumatoid arthritis. Terapevticheskii arkhiv. 68(5), 22-4.
